# Who Is Next? A Study on Victims of Financial Fraud in Japan

**DOI:** 10.3389/fpsyg.2021.649565

**Published:** 2021-07-02

**Authors:** Yoshihiko Kadoya, Mostafa Saidur Rahim Khan, Jin Narumoto, Satoshi Watanabe

**Affiliations:** ^1^School of Economics, Hiroshima University, Hiroshima, Japan; ^2^Department of Psychiatry, Graduate School of Medical Science, Kyoto Prefectural University of Medicine, Kyoto, Japan; ^3^Research and Education Center for Comprehensive Science, Akita Prefectural University, Akita, Japan

**Keywords:** financial fraud, victim, Japan, aged society, COVID-19 pandemic

## Abstract

Japan has seen an increase in the incidents of financial frauds over the last couple of decades. Although authorities are aware of the problem, an effective solution eludes them as fraudsters use innovative swindling methods and continually change the target group. Using a nationwide survey conducted by Hiroshima University, Japan, in 2020, this study investigated the socioeconomic and psychological profiles of victims of trending and special financial fraud such as fictitious billing fraud, loan guarantee fraud, and refund fraud. It was found that financial fraud victims' profiles are dissimilar at the aggregate and specific levels. At the specific level, victim profiles were diverse, that is, in fictitious billing fraud, loan guarantee fraud, and refund fraud cases. Males, married, and financially less satisfied people were more often victims of fictitious billing fraud; less anxious people were more likely victims of loan guarantee fraud; and older, asset-holding, and less-income-generating respondents were found to be victims of refund fraud. Our results also show some commonalities in the victims' profiles. For example, financially less-literate people were found to be more likely victims of fictitious billing fraud and loan guarantee fraud. Finally, respondents who lived with their family, those who did not have careful buying habits, and those who suffer from bouts of loneliness were found to be common victims of all types of special financial fraud. The results of our study suggest that a one-size-fits-all policy cannot effectively combat financial fraud.

## Introduction

### Background

Globally, incidents of “special” financial frauds, such as fictitious billing fraud, loan guarantee fraud, and refund fraud, have been on the rise over the last couple of decades causing concern among financial and legal authorities for the victims of these kinds of fraud (National Police Agency of Japan, 2009, 2017; Federal Trade Commission, 2013). However, authorities have been unable to find an effective solution to this problem, because fraudsters use innovative swindling methods and continually keep changing their target groups. For example, fraudsters are targeting older people for refund fraud, changing storylines for “it's me fraud,” recruiting new perpetrators and so on. Studies on financial frauds, at the aggregate level, and on common financial frauds, such as investment fraud, lottery fraud, online frauds, advance fee fraud, and others, are plenty. However, there are limited studies on trending and special financial frauds such as fictitious billing fraud, loan guarantee fraud, and refund fraud. This gap needs to be addressed by a comprehensive study because fraudsters target victims with certain backgrounds using innovative swindling packages. Lack of authentic information is one of the reasons for the lack of empirical studies on special financial frauds. Victims of financial frauds do not usually report these incidents because they are either unaware of the fraud when it happens, or they do not know where to report it, or do not report it at all because of the social embarrassment it involves (Ross and Smith, 2011; The Japan Times, 2019; Kadoya et al., 2020a; The United States Department of Justice, 2020). A nationwide study conducted by Hiroshima University, Japan, in 2020 comprehensively surveyed special financial frauds. The survey provides us an opportunity to investigate how people's demographic, socioeconomic, and psychological backgrounds make them probable victims of special financial frauds. We hypothesize that the profiles of victims of special financial frauds are different at the aggregate and specific levels; in other words, people of a certain demographic, socioeconomic, and psychological background are more vulnerable to a specific type of special financial fraud. Specifically, we hypothesize that males, older, married, those who live with family, less educated, less financially literate, unemployed, those who have higher household income and household assets, have myopic view, are less satisfied with their current financial condition, have less anxiety about future life, have less careful buying habits, have more trust on others, and feel lonely are more likely to become victims of financial frauds at the aggregate level. However, these factors are likely to have a different influence at the specific level because peoples' behavior, cognitive judgment, and lifestyle make them susceptible to a particular type of fraud. Kadoya et al. (2020a), a study closely related to ours, discussed the phenomena of financial scams in Japan and outlined victim profiles. However, the study was conducted at the aggregate level and the number of victims in the sample was limited. Our study is the first, to the best of our knowledge, to profile victims of special financial frauds. It confirms that victim profiles are specific to the type of financial fraud and that victim profiles should not be overgeneralized. Further, the sample size used is much larger than that of previous studies and covers the entire country.

### The Scenario of Fraud in Japan

Although Japan is a country known for safety and security, incidents around consumer and financial fraud are not uncommon. Fraudsters often devise swindling methods according to the social and economic backgrounds of the victims. Some of the common and globally evident frauds such as investment fraud like Ponzi schemes or Pyramid investments, deposit fraud, financial instrument fraud, telemarketing and mail fraud, lottery fraud, and online payment fraud are also evident in Japan. However, “it's me fraud” (in Japanese “ore ore sagi”), where fraudsters request family members to send money while impersonating their children or grandchildren, probably exceeds other types of fraud that are common in Japan (National Police Agency of Japan, 2019; Kadoya et al., 2020a). Moreover, the special financial fraud such as fictitious billing fraud, loan guarantee fraud, and refund fraud have been trending in Japan over the last decade (National Police Agency of Japan, 2017, 2019). The actual scenario of fraud is hard to understand because of the underreporting of fraud cases. In addition, sometimes victims are not aware of the legal procedure of reporting fraud. However, there are several laws and agencies to protect people from fraud in Japan. A number of provisions under the Japanese penal code are there to protect special frauds and misappropriation of fund (The Ministry of Justice, 2009). Besides, there are some provisions in the Financial Instruments and Exchange Act and Act on the Punishment of Organized Crime and the Control of Criminal Proceeds to combat special frauds. The National Police Agency of Japan is the leading anti-fraud authority in charge of reporting and investigating frauds. Moreover, the Japan Company Trust Organization and the Japan Anti-Fraud Organization also provide support to the victims of frauds. However, despite the efforts from law enforcing agencies, such as various awareness programs, the prevalence of telephone fraud, online payment fraud, and special financial frauds are difficult to contain (National Police Agency of Japan, 2019). A recent study showed that about 10.5% of the respondents experienced damage from special frauds in Japan and the total reported loss due to special frauds stands at 36.39 billion yen in 2018 (National Police Agency of Japan, 2019).

### Definitions, Classifications, and Incidence of Special Financial Frauds in Japan

Financial fraud involves deceiving people to gain money or assets through deceptive, misleading, and illegal financial transactions or investment projects. The United States Department of Justice (2020) defines fraud as deceiving people with the promise of goods, services, or financial benefits that are non-existent, were never intended to be provided, or were misrepresented. Victims of financial fraud experience not only financial losses but also major depression and other non-financial consequences (Button et al., 2010; Financial Industry Regulatory Authority, 2015). Several studies have been conducted on several investment frauds, such as Ponzi schemes, pyramid investments, and hedge fund related fraud that have shaken the financial world in the last couple of decades (Davis and Wilson, 2011; Bollen and Pool, 2012; Amoah, 2018). Over the years, authorities have initiated preventive measures against these kinds of fraud, making it difficult for fraudsters to replicate their methods. However, this has led to fraudsters improvising their techniques and perpetrating new types of financial fraud using innovative channels and targeting new groups of people. Globally, incidents of special financial fraud have become rampant with new groups targeted and at risk of becoming victims. Fictitious billing fraud is one such special financial fraud that has caused financial losses to many victims (National Police Agency of Japan, 2017; Flasher and Lamboy-Ruiz, 2019). In this type of fraud, fraudsters may send a false invoice, materially change an original invoice, mention their own address or bank accounts instead of that of the company, send an invoice multiple times, bill for unauthorized or unintentional membership, or bill for internet-related services such as website access, website hosting, or website development. A billing fraud may also involve unauthorized billing to customers for products or services, which they never agreed to purchase. A recent report in Japan shows the country incurred a financial loss of 13.74 billion yen in 2018 due to billing fraud, which was 7.7% higher than that of the previous year (The Japan Times, 2019). The National Police Agency of Japan (NPA) also reported that fictitious billing fraud was the most prevalent special financial fraud in Japan (National Police Agency of Japan, 2017). Another emerging special financial fraud is the loan guarantee fraud, wherein fraudsters stand guarantee for the sanction of a loan or credit card and, in return, collect upfront fees and sensitive information (Treece, 2020). Vozza (2020) identified several features of a loan guarantee fraud, such as guarantee for loan approvals, unspecified fees, and lenders, usually from different states, demanding immediate commitment and credit card information. In a loan guarantee fraud, fraudsters usually ask for credit card information or sensitive bank information as a requirement for loan disbursement and use that information to forge money at a later stage. Sometimes, they collect financial information in advance by hacking an account and using that information purportedly as victims' bankers. Fraudsters also offer unsolicited loans to victims personally or through email or telephone convincing them about the very lucrative terms and conditions of the loans. They may offer low interest rates and relax the requirement for collateral or creditworthiness, but pressurize the victim saying that the offer is available only for a short period. Fraudsters are sometimes so organized that they know that the victims have applied for loans and may even have access to victims' credit history. NPA reports that the financial value of loan fraud in 2016 was 700 million yen, which is a significant amount under the concurrent victim circumstances (National Police Agency of Japan, 2017). The last type of special financial fraud examined in this study is the refund fraud, wherein fraudsters impersonate as staff handling victims' tax, insurance, or other expenses and pretend to help victims get refund on expenses owed to them. Fraudsters convince victims that transfer of refund would be conducted through ATMs and usually instruct them on how to receive refunds over the phone while using ATMs. Victims often fail to understand that the instructions lead them to send money to fraudsters instead of receiving the refund into their account (National Police Agency of Japan, 2009). A recent study shows a rise in refund fraud in Japan, amounting to 1.43 billion yen in the first half of 2019 (National Police Agency of Japan, 2017; The Japan Times, 2018; Nippon.com, 2019). In refund fraud, perpetrators have even tried changing the mode of extortion. National Police Agency of Japan (2009) reports that perpetrators impersonated tax officials in the early cases of refund fraud. However, after 2007, they changed their tactics pretending to be social insurance officials handling medical expenses.

### Profiles of Victims of Financial Frauds

Understanding fraud victims' profiles has always been challenging because fraud incidents are under-reported and under-admitted (Deevy et al., 2012; The United States Department of Justice, 2020). Further, fraudsters frequently change their techniques of committing fraud as well as their target making the task more difficult. Studies have provided evidence on the emergence of new types of financial crimes (National Police Agency of Japan, 2009, 2017; Deevy et al., 2012) and explored the general trend of financial fraud associating them with the demographic, socioeconomic, and psychological backgrounds of the victims. Conventionally, financial crimes appear to be mostly targeted at older people as they are less technologically savvy, easy to convince, and sympathetic by nature (Button et al., 2014; Skiba, 2019; The Japan Times, 2019; Deliema et al., 2020; Kadoya et al., 2020a). Shao et al. (2019) found that the higher incidence of financial crimes among older people can be attributed to their lack of cognitive ability, emotional regulation and motivational changes, overly trusting nature, psychological vulnerability, social isolation, and lack of knowledge and information. Besides age, victims of financial fraud are mostly males (Button et al., 2014; Deliema et al., 2020), people living in metropolitan areas (The Japan Times, 2019), those dissatisfied with their current financial condition, and with a lower level of conscientiousness (Kadoya et al., 2020a). However, these general victim profiles fail to provide a reliable scenario for a specific financial fraud; previous studies have shown a diversity in the demographic and socioeconomic profiles across the different types of financial fraud. Schoepfer and Piquero (2009) found that other than indulging in risky behaviors and age, specific factors predicting fraud victimization varied across fraud types. National Police Agency of Japan (2009) reported that people over the age of 60 years were the primary victims of extortion fraud (it's me fraud), those below 40 were the primary victims of fictitious billing fraud, and those between 30 and 60 were primary victims of loan guarantee fraud. Moreover, the Federal Trade Commission (2007) reported that younger consumers were more likely to become victims of consumer fraud. Pak and Shadel (2011) found that males were more likely to be victims of investment fraud, while females were more likely to be victims of lottery fraud. Victims of investment fraud were found to have a higher income, while victims of lottery fraud were more likely to have a lower income. Ledbetter (2003) and Burton (2008) found that victims of investment fraud were more educated and showed financial literacy, while victims of lottery fraud were less educated and lacked financial literacy. Victims' diversity was seen in terms of marital status, race, education, financial literacy, openness, and others as well (Federal Trade Commission, 2007; Burton, 2008; Pak and Shadel, 2011; Deevy et al., 2012). This diversity evident in the demographic, socioeconomic, and psychological profiles of financial fraud victims shows the need for investigating victim profiles of special financial fraud on a case-by-case basis.

In section Data and Methods of this paper, data gathering and methods are described, followed by the empirical findings in section Empirical Findings, the discussion in section Discussion, and the conclusions in section Conclusion.

## Data and Methods

### Data

#### Participants

In this study, we use data from a nationwide online survey conducted in 2020 by Hiroshima University, Japan, based on a random sampling procedure where the minimum age of the prospective participants was 20 years. The study sample was drawn from one of the largest nationwide databases of the Nikkei Research Company of Japan. The survey considered all observations of equal strength and ensured proper representation from all socioeconomic backgrounds. The sample size used was 11,218, from which we identified individuals who had been victims of special financial fraud during the last 3 years before the survey.

#### Variables' Definitions

Victims of special financial fraud formed the dependent variable. If respondents answered “yes” on being asked if they had experienced any financial fraud, such as fictitious billing fraud, loan guarantee fraud, or refund fraud, during the last 3 years before the survey (also included those who experienced financial fraud but were able to avoid damage), we considered them at the aggregate and specific levels.

Participants' demographic, socioeconomic, and psychological backgrounds formed the independent variables of the study. Based on the findings of previous studies on the association between demographic backgrounds and the probability of being victims of financial fraud (Van Wyk and Mason, 2001; Lee, 2005; Federal Trade Commission, 2007; Deevy et al., 2012; Skiba, 2019; Deliema et al., 2020; Kadoya et al., 2020a), we included several demographic factors such as gender, age, marital status, and living with family. Earlier studies have also proved that people's socioeconomic status was associated with the probability of becoming victims of financial fraud (Federal Trade Commission, 2004, 2007; Lee, 2005; Burton, 2008; Pak and Shadel, 2011; Ross and Smith, 2011; Deevy et al., 2012; Kadoya et al., 2020a). Along with commonly used socioeconomic backgrounds, such as education, household income, household balance of financial assets, residential status, and employment status, we also included financial literacy as an independent variable in our study because financial literacy was found to be related to financial fraud (Ledbetter, 2003; Burton, 2008). To measure financial literacy, we followed the methodology of Lusardi and Mitchell (2008), which has been widely adopted in the existing literature (Fornero and Monticone, 2011; Lusardi and Mitchell, 2011, 2014; Kadoya and Khan, 2018, 2020; Kadoya et al., 2018, 2020a; Watanapongvanich et al., 2020). Finally, we included several variables related to respondents' psychological characteristics because previous studies found an association between peoples' psychology and consumer fraud (Shover et al., 2003; Office of Fair Trading, 2006; Schoepfer and Piquero, 2009; Kadoya et al., 2020a). The psychological variables used in this study were myopic view, level of financial satisfaction, anxiety about life in old age, careful buying habits, trust in others, and loneliness. Table 1 provides the definitions and measurements of all variables.

**Table 1:** Definition and measurement of variables.

**Dependent variable**	
Special financial frauds	Whether participants have experienced special financial frauds such as fictitious billing fraud, loan guarantee fraud, or refund fraud in the last 3 years of conducting the survey (also included those who experienced financial fraud but were able to avoid damage). Respondents' experience about special financial frauds were measured from the following question: “Which type of bank transfer swindle did you experience?” The options included “Fictitious billing fraud,” “Loan guarantee fraud,” “Refund fraud,” “I don't know or don't want to answer.” The dependent variable is binary in nature where 1 = victim of special financial frauds such as fictitious billing fraud, loan guarantee fraud, and refund fraud, 0 = otherwise
**Independent variables**	
Gender	Gender of respondents. 1 = male, 0 = female
Age	Age of respondents in years
Marital status	Marital status of respondents where 1 = married, 0 = otherwise
Living with family	1 = respondents living with family, 0 = otherwise
Education	Years of education completed by respondents
Financial literacy	Financial literacy measures respondents' ability to understand basic financial calculations, inflation, and risks of financial securities. Following questions were asked to respondents: 1. Suppose you had ¥100 in your savings account, the interest rate is 2% per year, and you never withdraw money or interest payments. After 5 years, how much would you have in this account? □ More than ¥102 □ Exactly ¥102 □ Less than ¥102 □ Do not know □ Refuse to answer 2. Assume that the interest rate on your savings account is 1% per year and inflation is 2% per year. After 1 year, how much would you be able to buy with the money in this account? □ More than today □ Exactly the same □ Less than today □ Do not know □ Refuse to answer 3. Indicate whether the following statement is true or false: “Buying a company stock usually provides a safer return than a stock mutual fund.” □ True □ False □ Do not know □ Refuse to answer
Employment status	Employment status where 1 = currently employed, 0 = otherwise
Household income	Annual household income in yen
Household assets	Household balance of financial assets in yen
Myopic view	Respondents' perceptions about the future, which was measured by the following statement: “Since the future is uncertain, it is a waste of time thinking about it” (5 being completely agree and 1 being completely disagree).
Financial satisfaction	Respondents' current level of financial satisfaction, which was measured by the following statement: “I am happy with my financial status” (5 being completely agree and 1 being completely disagree).
Anxiety	Respondents' anxiety about life in old age, which was measured by the following statement: “I have anxieties about my life after I turn 65” (5 being the highest and 1 being the lowest).
Careful spending habit	Respondents' carefulness in spending, which is measured by the following statement: “I think carefully before buying anything” (5 being the highest and 1 being the lowest).
Trust	Respondents' trust in other people, which was measured by the following statement: “In general, most people are trustworthy” (5 being completely agree and 1 being completely disagree).
Loneliness	The extent to which respondents feel loneliness. Respondents' loneliness was measured by the following question: “How often do you feel lonely” (1 being never and 5 being often or always).

#### Descriptive Statistics

Table 2 presents the descriptive statistics of the variables used in this study. Results show that 4.97% (SD = 21.72%) of the respondents were victims of at least one type of special financial fraud in the last 3 years before the survey. The fraud prevalence rate seems substantial under the scenario of underreporting of fraud cases as 558 out of 11,218 respondents experienced special financial frauds. Most respondents were victims of fictitious billing fraud (Mean = 3.44%, SD = 18.23%) followed by loan guarantee fraud (Mean = 1.16%, SD = 10.70%) and refund fraud (Mean = 0.87%, SD = 9.26%). Demographic profiles of respondents show that 61.19% are males and their average age was 47.59 years (SD = 14.36 years). Further, 82.38% (SD = 38.10%) of the respondents were married and 80.66% (SD = 39.50%) lived with their family. Socioeconomic status of the respondents shows that, on an average, they attained 14.90 years of education (SD = 2.08 years), had moderate financial literacy (Mean = 0.6557, SD = 0.3565), an annual household income of 5,480,000 yen (SD = 1,092,500), and an average balance of 7,215,250 yen (SD = 4,626,250) as assets. Respondents' psychological characteristics show that they are moderately myopic about the future (Mean = 2.58, SD = 1.01), moderately satisfied with their current financial condition (Mean = 2.70, SD = 1.10), are anxious about their life in old age (Mean = 3.72, SD = 1.18), are careful buyers (Mean = 4.0414, SD = 0.9854), have a moderate level of trust in others (Mean = 2.81, SD = 0.95), and are moderately lonely (Mean = 2.87, SD = 1.19). The demographic, socio-economic, and psychological characteristics of respondents are consistent with previous studies (Kadoya et al., 2021; Khan et al., 2021; Ono et al., 2021; Watanapongvanich et al., 2021).

**Table 2:** Descriptive statistics.

**Variable**	**Obs**.	**Mean**	**Std. dev**.	**Min**.	**Max**.
Special financial frauds	11,218	0.0497	0.2172	0	1
Fictitious billing fraud	11,218	0.0344	0.1823	0	1
Loan guarantee fraud	11,218	0.0116	0.1070	0	1
Refund fraud	11,218	0.0087	0.0926	0	1
Gender	11,218	0.6119	0.4873	0	1
Age	11,218	47.5869	14.3632	20	92
Marital status	11,218	0.8238	0.3810	0	1
Living with family	11,218	0.8066	0.3950	0	1
Education	11,218	14.9001	2.0776	9	21
Financial literacy	11,218	0.6557	0.3565	0	1
Employment status	11,218	0.6658	0.4717	0	1
Household income (in thousand yen)	11,218	5,489.0000	1,092.5000	1,000	20,000
Household assets (in thousand yen)	11,218	7,215.2500	4,626.2500	2,500	100,000
Myopic view	11,218	2.5828	1.0067	1	5
Financial satisfaction	11,218	2.6994	1.0965	1	5
Anxiety	11,218	3.7199	1.1745	1	5
Careful spending habit	11,218	4.0414	0.9854	1	5
Trust	11,218	2.8141	0.9448	1	5
Loneliness	11,218	2.8682	1.1887	1	5

*Obs., observation; Std. dev., standard deviation; Min., minimum; Max., maximum*.

Table 3 presents a detailed description of special financial frauds such as fictitious billing fraud, loan guarantee fraud, and refund fraud based on important demographic, socioeconomic, and psychological variables. We found a uniformity in the victim profiles based on gender, age, buying habit, and loneliness. Results show that, for all types of financial fraud, males were more likely to be victims than females; also younger (lower than 40 years of age) and older (more than 65 years of age) respondents were likely to become victims than middle aged (between 40 and 65 years) respondents; less careful buyers were more likely to be victims than their careful counterparts; and, finally, lonely respondents (respondents who felt lonely at least occasionally) were likely to be victims than their less lonely counterparts (who never or hardly felt lonely). However, victim profiles were not so uniform across marital status, education, household income, household balance of financial assets, and employment status. Unmarried respondents were more susceptible to fall prey to billing fraud than their married counterparts, who were more likely to be victims of loan fraud and refund fraud than unmarried respondents. Less educated respondents were more likely victims of fictitious billing fraud than respondents who were highly or moderately educated; but highly educated respondents were more likely victims of loan guarantee fraud while moderately educated respondents were victims of refund fraud. Lower-income respondents were more likely to be victims of billing fraud and refund fraud than their higher-income counterparts, but respondents in the higher-income bracket were more likely victims of loan guarantee fraud. In terms of financial assets, those with a lower balance were victims of fictitious billing while those with a higher balance were victims of loan guarantee fraud and refund fraud. Unemployed respondents were more likely to be victims of fictitious billing fraud and refund fraud, while employed respondents were mostly victims of loan guarantee fraud.

**Table 3:** Detailed description of special financial frauds based on important variables.

**Variables**		**Fictitious billing fraud**	**Loan guarantee fraud**	**Refund fraud**
Gender	Female	0.0312 (0.1740)	0.0096 (0.0978)	0.0069 (0.0827)
	Male	0.0364 (0.1874)	0.0128 (0.1125)	0.0098 (0.0983)
Age	<40	0.0353 (0.1846)	0.0133 (0.1147)	0.0076 (0.0868)
	40–65	0.0310 (0.1733)	0.0101 (0.0998)	0.0060 (0.0774)
	>65	0.0463 (0.2101)	0.0133 (0.1147)	0.0224 (0.1481)
Marital status	Married	0.0330 (0.1787)	0.0119 (0.1085)	0.0089 (0.0938)
	Not married	0.0410 (0.1983)	0.0101 (0.1001)	0.0076 (0.0868)
Education	<12	0.0392 (0.1948)	0.0000 (0.0000)	0.0065 (0.0808)
	12–16	0.0351 (0.1841)	0.0112 (0.1053)	0.0090 (0.0946)
	>16	0.0265 (0.1606)	0.0173 (0.1304)	0.0051 (0.0712)
Employment status	Employed	0.0333 (0.1795)	0.0125 (0.1109)	0.0072 (0.0847)
	Not employed	0.0365 (0.1877)	0.0099 (0.0989)	0.0115 (0.1065)
Household income	Below average	0.0369 (0.1884)	0.0110 (0.1045)	0.0090 (0.0945)
	Above average	0.0317 (0.1752)	0.0122 (0.1098)	0.0083 (0.0905)
Household assets	Below average	0.0355 (0.1850)	0.0106 (0.1025)	0.0063 (0.0791)
	Above average	0.0332 (0.1791)	0.0127 (0.1121)	0.0114 (0.1061)
Careful spending habit	Mostly careful	0.0331 (0.1790)	0.0069 (0.0829)	0.0084 (0.0910)
	Mostly careless	0.0352 (0.1842)	0.0144 (0.1191)	0.0088 (0.0935)
Loneliness	Mostly lonely	0.0409 (0.1982)	0.0148 (0.1207)	0.0095 (0.0970)
	Hardly lonely	0.0257 (0.1581)	0.0073 (0.0851)	0.0075 (0.0863)

### Methods

We used logit regression models to investigate how respondents' demographic, socioeconomic, and psychological backgrounds are associated with special financial fraud. Fraud victimization is measured both at the aggregate and at the specific levels. The aggregate indicator identifies whether respondents have been victims of any type of special financial fraud, while the specific indicator consider specific special financial fraud. We formulated four models to estimate factors associated with special financial fraud at the aggregate level, that is, respondents who were victims of special financial frauds of any type (Model 1), and at specific level, that is, respondents who were victims of fictitious billing fraud (Model 2), loan guarantee fraud (Model 3), and refund fraud (Model 4). In all models, the dependent variables are binary in nature, taking the value 1 if respondents have been victims of special financial fraud and 0 if otherwise. We used demographic (gender, age, marital status, and living with family), socioeconomic status (education, financial literacy, employment status, household income, and household balance of financial assets), and psychological and behavioral factors (myopic view, financial satisfaction, anxiety, spending habit, trust, and loneliness) as explanatory variables in all models. The model specifications are as follows:

(1) $$\begin{array}{l} \text{special}\;\text{financial}\;\text{fraud}{\text{s}}_{\text{i}}\;\left(1=\text{victims}\;\text{of}\;\text{special}\;\text{financial}\;\text{fraud}\;\text{and}\;0=\text{non}-\text{victims}\right)=\beta_{0}+\beta_{1}\text{gende}{\text{r}}_{\text{i}}\;+\beta_{2}\text{ag}{\text{e}}_{\text{i}}\\ +\beta_{3}\text{marital}\;\text{statu}{\text{s}}_{\text{i}}+\beta_{4}\text{living}\;\text{with}\;\text{famil}{\text{y}}_{\text{i}}+\beta_{5}\text{educatio}{\text{n}}_{\text{i}}+\beta_{6}\text{financial}\;\text{literac}{\text{y}}_{\text{i}}+\beta_{7}\text{employment}\;\text{statu}{\text{s}}_{\text{i}}\\ +\beta_{8}\text{household}\;\text{incom}{\text{e}}_{\text{i}}+\beta_{9}\text{household}\;\text{asset}{\text{s}}_{\text{i}}+\beta_{10}\text{myopic}\;\text{vie}{\text{w}}_{\text{i}}+\beta_{11}\text{financial}\;\text{satisfactio}{\text{n}}_{\text{i}}+\beta_{12}\text{anxiet}{\text{y}}_{\text{i}}\\ +\beta_{13}\text{careful}\;\text{buying}\;\text{habi}{\text{t}}_{\text{i}}+\beta_{14}\text{trus}{\text{t}}_{\text{i}}+\beta_{15}\text{lonelines}{\text{s}}_{\text{i}}+\varepsilon_{\text{i}} \end{array}$$

(2) $$\begin{array}{l} \text{finctitious}\;\text{billing}\;\text{frau}{\text{d}}_{\text{i}}\;\left(1=\text{victims}\;\text{of}\;\text{fictitious}\;\text{billing}\;\text{fraud}\;\text{and}\;0=\text{non}-\text{victims}\right)=\;\beta_{0}+\beta_{1}\text{gende}{\text{r}}_{\text{i}}\;+\beta_{2}\text{ag}{\text{e}}_{\text{i}}\\ +\beta_{3}\text{marital}\;\text{statu}{\text{s}}_{\text{i}}+\beta_{4}\text{living}\;\text{with}\;\text{famil}{\text{y}}_{\text{i}}+\beta_{5}\text{educatio}{\text{n}}_{\text{i}}+\beta_{6}\text{financial}\;\text{literac}{\text{y}}_{\text{i}}+\beta_{7}\text{employment}\;\text{statu}{\text{s}}_{\text{i}}\\ +\beta_{8}\text{household}\;\text{incom}{\text{e}}_{\text{i}}+\beta_{9}\text{household}\;\text{asset}{\text{s}}_{\text{i}}+\beta_{10}\text{myopic}\;\text{vie}{\text{w}}_{\text{i}}+\beta_{11}\text{financial}\;\text{satisfactio}{\text{n}}_{\text{i}}+\beta_{12}\text{anxiet}{\text{y}}_{\text{i}}\\ +\beta_{13}\text{careful}\;\text{buying}\;\text{habi}{\text{t}}_{\text{i}}+\beta_{14}\text{trus}{\text{t}}_{\text{i}}+\beta_{15}\text{lonelines}{\text{s}}_{\text{i}}+\varepsilon_{\text{i}} \end{array}$$

(3) $$\begin{array}{l} \text{loan}\;\text{guarantee}\;\text{frau}{\text{d}}_{\text{i}}\;\left(1=\text{victims}\;\text{of}\;\text{loan}\;\text{guarantee}\;\text{fraud}\;\text{and}\;0=\text{non}-\text{victims}\right)=\beta_{0}+\beta_{1}\text{gende}{\text{r}}_{\text{i}}\;+\beta_{2}\text{ag}{\text{e}}_{\text{i}}\\ +\beta_{3}\text{marital}\;\text{statu}{\text{s}}_{\text{i}}+\beta_{4}\text{living}\;\text{with}\;\text{famil}{\text{y}}_{\text{i}}+\beta_{5}\text{educatio}{\text{n}}_{\text{i}}+\beta_{6}\text{financial}\;\text{literac}{\text{y}}_{\text{i}}+\beta_{7}\text{employment}\;\text{statu}{\text{s}}_{\text{i}}\\ +\beta_{8}\text{household}\;\text{incom}{\text{e}}_{\text{i}}+\beta_{9}\text{household}\;\text{asset}{\text{s}}_{\text{i}}+\beta_{10}\text{myopic}\;\text{vie}{\text{w}}_{\text{i}}+\beta_{11}\text{financial}\;\text{satisfactio}{\text{n}}_{\text{i}}+\beta_{12}\text{anxiet}{\text{y}}_{\text{i}}\\ +\beta_{13}\text{careful}\;\text{buying}\;\text{habi}{\text{t}}_{\text{i}}+\beta_{14}\text{trus}{\text{t}}_{\text{i}}+\beta_{15}\text{lonelines}{\text{s}}_{\text{i}}+\varepsilon_{\text{i}} \end{array}$$

(4) $$\begin{array}{l} \text{refund}\;\text{frau}{\text{d}}_{\text{i}}\;\left(1=\text{victims}\;\text{of}\;\text{refund}\;\text{fraud}\;\text{and}\;0=\text{non}-\text{victims}\right)=\beta_{0}+\beta_{1}\text{gende}{\text{r}}_{\text{i}}\;+\beta_{2}\text{ag}{\text{e}}_{\text{i}}\\ +\beta_{3}\text{marital}\;\text{statu}{\text{s}}_{\text{i}}+\beta_{4}\text{living}\;\text{with}\;\text{famil}{\text{y}}_{\text{i}}+\beta_{5}\text{educatio}{\text{n}}_{\text{i}}+\beta_{6}\text{financial}\;\text{literac}{\text{y}}_{\text{i}}+\beta_{7}\text{employment}\;\text{statu}{\text{s}}_{\text{i}}\\ +\beta_{8}\text{household}\;\text{incom}{\text{e}}_{\text{i}}+\beta_{9}\text{household}\;\text{asset}{\text{s}}_{\text{i}}+\beta_{10}\text{myopic}\;\text{vie}{\text{w}}_{\text{i}}+\beta_{11}\text{financial}\;\text{satisfactio}{\text{n}}_{\text{i}}+\beta_{12}\text{anxiet}{\text{y}}_{\text{i}}\\ +\beta_{13}\text{careful}\;\text{buying}\;\text{habi}{\text{t}}_{\text{i}}+\beta_{14}\text{trus}{\text{t}}_{\text{i}}+\beta_{15}\text{lonelines}{\text{s}}_{\text{i}}+\varepsilon_{\text{i}} \end{array}$$

As explanatory variables can be potentially multicollinear (e.g., highly educated respondents could have high financial literacy, or individuals with higher financial assets could have more household income), we conducted correlation and multicollinearity tests in all models (the results are not reported here to save on space but are available as a Supplementary Material). The results show that multicollinearity between the variables is not significant (the variance inflation factors of the explanatory variables are well below 10), suggesting that the independent effects of explanatory variables on the probability of being victims of special financial fraud are not biased. The correlation matrix shows a weak relationship between the explanatory variables (<0.50).

## Empirical Findings

Since we investigated how respondents' demographic, socioeconomic, and psychological backgrounds are associated with special financial fraud at the aggregate and specific levels, it was important to isolate victims of a specific special financial fraud from those of other types of fraud. We measured the extent of joint victimization in special financial fraud. In 13 cases, respondents were victims of all types of special financial fraud; in 29 cases, they were victims of fictitious billing fraud and loan guarantee fraud; in 25 cases, respondents were victims of fictitious billing fraud and refund fraud; and in 15 cases, respondents were victims of loan guarantee fraud and refund fraud. We considered all respondents when we estimated how demographic, socioeconomic, and psychological backgrounds are associated with special financial fraud at the aggregate level. However, we excluded cases of joint victimization when estimating the association between demographic, socioeconomic, and psychological backgrounds and a specific type of special financial fraud. The exclusion of respondents who were victims of multiple financial fraud allowed us to provide clearer evidence of the profile of victims of a specific type of special financial fraud. Model 1 shows the regression results for victims of special financial fraud at the aggregate level, whereas Model 2, Model 3, and Model 4 represent the regression results for victims of fictitious billing fraud, loan guarantee fraud, and refund fraud, respectively. In all models, LR chi^2^ is statistically significant, indicating the validity of the models and that some variables used in the models are significantly associated with financial fraud.

Table 4 shows the results of the logit regression models. The results of Model 1 show that gender, age, living with family, household balance of financial assets, and feelings of loneliness are positively correlated to the probability of becoming victims of financial fraud at the aggregate level at 5, 10, 1, 10, and 1% level of significance, respectively, while marital status, financial literacy, myopic view, and careful buying habit are negatively correlated at 5, 1, 10, and 1% level of significance, respectively. The results indicate that for a one-unit increase in gender (going from female to male), age, living with family, household balance of financial assets, and feelings of loneliness, the log-odds of becoming victims of special financial frauds are increased by 0.2689, 0.0065, 0.6852, 0.0322, and 0.2025, respectively, while for a one-unit increase in marital status, financial literacy, myopic view, and careful buying habit, the log-odds of becoming victims of special financial frauds are decreased by 0.2560, 0.4678, 0.0765, and 0.2342, respectively. Thus, respondents who are males, older, currently living with family, with a higher household balance of financial assets, not currently married, less financially literate, less myopic, less careful buyers, and feel lonely most of the time are more likely to be victims of financial fraud. However, education, employment status, household income, current level of financial satisfaction, anxiety about life in old age, and trust showed no correlation to the probability of being victims of financial fraud. The results of Model 2 show that gender, living with family, and the feelings of loneliness are positively correlated to the probability of being victims of fictitious billing fraud at 10, 1, and 1% level of significance, respectively, while marital status, financial literacy, financial satisfaction, and careful buying habits show a negative correlation at 5, 1, 10, and 10% level of significance, respectively. The results indicate that for a one-unit increase in gender (going from female to male), living with family, and the feelings of loneliness, the log-odds of becoming victims of special financial frauds are increased by 0.2406, 0.6785, and 0.1564, respectively, while for a one-unit increase in marital status, financial literacy, financial satisfaction, and careful buying habits, the log-odds of becoming victims of special financial frauds are decreased by, 0.3389, 0.4472, 0.0988, and 0.0932, respectively. Thus, male, currently living with family, not currently married, less literate, less satisfied with the current financial condition, less careful in buying, and feeling lonely most of the time are more likely to be victims of fictitious billing fraud. However, age, education, employment status, household income, household assets, myopic view, anxiety, and trust showed no association with the probability of such respondents becoming victims of fictitious billing fraud. Results of Model 3 show that living with family and the feeling lonely are positively correlated to becoming victims of loan guarantee fraud at 5 and 1% level of significance, respectively, while financial literacy, anxiety, and careful buying show a negative correlation at 5, 5, and 1% level of significance, respectively. The results indicate that for a one-unit increase in living with family and the feeling lonely, the log-odds of becoming victims of special financial frauds are increased by 0.8376 and 0.2839, respectively, while for a one-unit increase in financial literacy, anxiety, and careful buying habits, the log-odds of becoming victims of special financial frauds are decreased by 0.6135, 0.2066, and 0.5296, respectively. Thus, respondents currently living with their family, those less financially literate, less careful buyers, less anxious about life in old age, and those who feel lonely most of the time are more likely to be victims of loan guarantee fraud. However, gender, age, marital status, education, employment status, household income, household assets, myopic view, current level of financial satisfaction, and trust are not found to be associated with the probability of becoming victims of loan guarantee fraud. Finally, results of Model 4 show that age, living with family, household balance of financial assets, and feeling lonely are positively correlated to becoming victims of refund fraud at 5, 5, 10, and 10% level of significance, respectively, while household income and careful buying show negative correlation at 10 and 1% level of significance, respectively. The results indicate that for a one-unit increase in age, living with family, household balance of financial assets, and feeling lonely, the log-odds of becoming victims of special financial frauds are increased by 0.0237, 1.035, 0.0947, and 0.1871, respectively, while for a one-unit increase in household income and careful buying habits, the log-odds of becoming victims of special financial frauds are decreased by 0.1369 and 0.3787, respectively. Thus, older respondents, those living with family, those less careful about buying, those with higher household balance of financial assets, less household income, and feel lonely are more likely to be victims of refund fraud. However, gender, marital status, education, financial literacy, employment status, current level of financial satisfaction, anxiety about life in old age, and trust did not show any association with the probability of becoming victims of financial fraud.

**Table 4:** Regression results.

**Variable**	**Model 1 (Financial frauds at the aggregate level)**	**Model 2 (Fictitious billing fraud)**	**Model 3 (Loan guarantee fraud)**	**Model 4 (Refund fraud)**
Gender	0.2689 (2.39)^**^	0.2406 (1.76)^*^	0.1353 (0.52)	0.2964 (0.92)
Age	0.0065 (1.67)^*^	0.0049 (1.03)	0.0035 (0.39)	0.0237 (2.13)^**^
Marital status	−0.2560 (−2.01)^**^	−0.3389 (−2.23)^**^	−0.1616 (−0.52)	−0.1338 (−0.35)
Living with family	0.6852 (4.77)^***^	0.6785 (3.91)^***^	0.8376 (2.36)^**^	1.035 (2.15)^**^
Education	−0.0053 (−0.23)	−0.0162 (−0.58)	0.0322 (0.61)	−0.0354 (−0.56)
Financial literacy	−0.4678 (−3.47)^***^	−0.4472 (−2.71)^***^	−0.6135 (−1.99)^**^	0.0868 (0.22)
Employment status	−0.0200 (−0.18)	−0.0142 (−0.10)	0.3213 (1.17)	−0.2441 (−0.80)
Household income	−0.0411 (−1.54)	−0.0406 (−1.20)	−0.0259 (−0.45)	−0.1369 (−1.81)^*^
Household assets	0.0322 (1.65)^*^	0.0073 (0.29)	0.0480 (1.09)	0.0947 (1.82)^*^
Myopic view	−0.0765 (−1.66)^*^	−0.0312 (−0.56)	−0.0928 (−0.87)	−0.1881 (−1.42)
Financial satisfaction	−0.0244 (−0.51)	−0.0988 (−1.67)^*^	0.1556 (1.40)	0.0863 (0.62)
Anxiety	−0.0639 (−1.53)	−0.0411 (−0.79)	−0.2066 (−2.26)^**^	−0.0477 (−0.41)
Careful spending habit	−0.2342 (−5.42)^***^	−0.0932 (−1.68)^*^	−0.5296 (−5.78)^***^	−0.3787 (−3.27)^***^
Trust	−0.0157 (−0.32)	−0.0144 (−0.24)	0.0249 (0.22)	0.1789 (1.27)
Loneliness	0.2025 (5.17)^***^	0.1564 (3.26)^***^	0.2839 (3.11)^***^	0.1871 (1.69)^*^
Cons	−2.4883 (−4.93)^***^	−2.9752 (−4.75)^***^	−4.3959 (−3.84)^***^	−5.6610 (−3.87)^***^
Obs.	11,218	11,189	11,187	11,191
LR Chi^2^	122.90^***^	55.78^***^	82.04^***^	55.37^***^
Pseudo *R*^2^	0.0277	0.0176	0.0724	0.0651
Log likelihood	−2,153.9965	−1,553.2012	−525.5523	−397.2996

*The z values in parentheses*.

***, **, and * represent significance at the 1, 5, and 10% levels, respectively.

The results show that profiles of victims of financial fraud at the aggregate and specific levels are not the same. Diversity in victims' profiles is found at the specific level, that is, among fictitious billing fraud, loan guarantee fraud, and refund fraud. Respondents who are male, married, and less satisfied with the current financial conditions were only found to be victims of fictitious billing fraud while those less anxious about life in old age were only found to be victims of refund fraud; older respondents, those with higher household balance of financial assets, and less household income were only victims of refund fraud. We also observe some commonalities in victim profiles across the types of special financial fraud. For example, respondents who are financially less literate are more likely to be victims of fictitious billing fraud and loan guarantee fraud. Finally, respondents who live with their family, do not have careful buying habits, and feel lonely are found to be common victims of all types of special financial fraud.

## Discussion

The increasing cases of financial fraud in Japan requires investigating factors associated with potential victims so that protective measures can be implemented to combat fraud. The widespread emergence of commercial transactions on online platforms, transactions from distant places, increasing interactions with unknown and unsolicited people, and others have contributed to the rise in financial fraud (National Police Agency of Japan, 2017, 2019). However, studies are lacking on who are potentially at risk of becoming victims of social financial fraud in Japan, such as fictitious billing fraud, loan guarantee fraud, and refund fraud. In this background, we investigated how demographic, socioeconomic, and psychological factors are associated with those who are likely to become victims of such special financial fraud. Our results show both diversity and commonality in victims' profiles in fictitious billing fraud, loan guarantee fraud, and refund fraud.

### Diversity in Victims' Profiles Among the Types of Special Financial Fraud

Although people's demographic orientation has been considered in several studies on consumer fraud, there has been mixed evidence on its association with their probability of becoming victims of consumer and financial fraud (Federal Trade Commission, 2004, 2007, 2013; Pak and Shadel, 2011; Kadoya et al., 2020a). Our study shows clear evidence of diversity in victims' demographic backgrounds across the types of financial fraud, suggesting that different groups of people are the usual targets for different types of financial fraud. Males and those not currently married were found to be more likely victims of fictitious billing fraud. We argue that such respondents more likely engage in commercial transactions and risky buying behavior, thus becoming victims of fictitious billing fraud. Although we expected a positive association, the negative association between fictitious billing fraud and marital status could be the result of more risky buying behavior of unmarried people. However, no association was seen between gender and marital status regarding loan guarantee fraud and refund fraud. Previous studies also found that gender was associated with some types of financial fraud, but not all (Pak and Shadel, 2011). Pak and Shadel (2011) found that males were more likely to be victims of investment fraud, while females were more likely to be victims of lottery fraud. Diversity has been reported in the relationship between marital status and victims of fraud. Sauer and Pak (2007), Burton (2008), and Pak and Shadel (2011) showed that investment fraud victims are more likely to be married, but lottery fraud victims were more likely to be widowed or divorced. Apart from gender and marital status, our results show that older people are more likely to victims of refund fraud, but no such conclusion can be drawn for fictitious billing fraud and loan guarantee fraud. We argue that fraudsters target elderly people because they are less technologically savvy and less aware of financial transactions. It should be noted that most of the refund crimes are conducted through bank transfers, where fraudsters instruct victims to follow procedures suggested by them. Older people fail to understand that the instructions they are following are in fact transferring funds from their account rather than being deposited. Diversity has also been reported in the association between age and fraud victims across types of fraud. Lokanan and Liu (2020) found that investors aged 60 years and above are more likely to be victims of financial frauds. Lokanan (2014) further argued that people who are retired and have limited investment knowledge are the most vulnerable to investment fraud. Federal Trade Commission (2013) reports that people aged between 55 and 74 years were more likely to be victims of prize promotion fraud. Ross and Smith (2011) found that respondents aged 65 years or older were more likely to be victims of some advance fee fraud, and Sauer and Pak (2007) found that victims of financial fraud were, on average, 55 years of age. However, studies of the Federal Trade Commission (2004, 2007) showed that older people were less likely to be victims of consumer fraud unlike their younger counterparts.

Our results show diversity in the socioeconomic backgrounds of victims who are likely to be targeted by the different types of special financial fraud. Those with lesser household income and those with a higher balance in terms of financial assets are likely victims of refund fraud. Thus, wealthy people may become targets, probably because of their economic capacity. However, a higher household balance of assets does not indicate their current income capacity. Therefore, people with a low household income can be attracted to refund proposals used by fraudsters. Our findings are consistent with those of earlier studies proving that victims of financial fraud are largely from low-income groups (Ross and Smith, 2011).

Diversity is also observed in the psychological characteristics of victims of the various types of financial fraud. People currently less satisfied with their financial condition are more likely to be victims of fictitious billing fraud and engage in risky transactions, which can attract the attention of fraudsters. Schoepfer and Piquero (2009) provided evidence that indulging in risky behavior made people susceptible to fraud victimization. Such persons are also less knowledgeable about financial matters (Murphy, 2013; Kadoya et al., 2020b) and the terms and conditions associated with products and services. The findings and arguments of this study are consistent with that from Consumer Fraud Research Group (2006) and Kadoya et al. (2020a), proving a link between financial dissatisfaction and fraud victimization. Our study further finds that people who are less anxious about life in their old age are more likely to be victims of loan guarantee fraud. Lack of understanding about the lending process, relying on unsolicited sources for lending, susceptible to persuasion, impatience, and lack of self-control are the principal reasons for loan guarantee fraud victimization. We argue that when people are not much concerned about their future, they tend to show impulsivity, lack cognition, and can be easily persuaded to believe in illegal means, making them susceptible to loan-guarantee fraud. Previous studies have provided evidence that impulsivity, cognitive inability, and susceptible to persuasion make people likely victims of fraud (AARP, 2003; Ledbetter, 2003).

In addition to finding victims' demographic, socio-economic, and psychological characteristics, it is important to explain why victims' profiles differ across the types of financial frauds. Overall, our results suggest not everyone faces the same risk of becoming a victim of fraud; instead, people's behavioral pattern, cognitive ability, and lifestyle are related to their risk of experiencing special financial frauds. Previous studies also found that the behavior of consumers (Holtfreter et al., 2008) and their cognitive judgments (Office of Fair Trading, 2006) are determining factors of fraud victimization. Although Pak and Shadel (2011) attributed differences in the findings of previous studies to methodological issues, we argue that factors related to culture could be responsible for such differences too. People have different orientation toward family, society, technology, and trust, as well as different levels of cognitive ability and motivation; and these are responsible for differences in the likelihood of victimization fraud across different countries.

### Commonality in Victims' Profiles Among the Types of Special Financial Fraud

We also found commonality in victims' profiles for being targets of special financial fraud. People who are financially less literate are more likely to be victims of fictitious billing fraud and loan guarantee fraud as financial literacy is a measure of people's understanding of financial calculations and the implications of financial and economic issues on financial transactions. Financial literacy is associated with people's higher cognitive ability, enabling them to critically evaluate products and services. Thus, people with less financial literacy may be unable to verify terms and conditions relevant to purchase of goods and services or the fee and charges attached to lending products. Financial literacy has been considered a factor in people being targets of some types of fraud. Sauer and Pak (2007) and Burton (2008) found that investment fraud victims were more financially literate, but lottery fraud victims less so. Federal Trade Commission (2013) provided evidence that people with limited numeric skills are more likely to be victims of some forms of consumer fraud.

The three most consistent factors associated with all types of financial fraud are living with family, careful buying habits, and a sense of loneliness. In our study, victims of fictitious billing fraud, loan guarantee fraud, and refund fraud were respondents living with family. The special financial fraud considered in this study has a family orientation; fictitious billing fraud is associated with frequent purchase of goods and services, loan guarantee fraud is associated with the sudden need for funds, and refund fraud is associated with refund of family expenses for tax, insurance, medical bill, etc. Higher commercial and financial transactions make victims visible and accessible, which are necessary conditions for fraud victimization (Felson and Clarke, 1998). It is easy for fraudsters to trap someone who has family members or to approach potential victims through their family members (Vickstorm, 2018; Federal Trade Commission, 2019). Careful buying habits also reflects respondents' sense of caution and awareness about commercial transactions. Not verifying offers on products and services, not checking transaction documents, and not being cautious in online dealings are characteristics of victims of financial fraud. Previous studies support this argument that lack of caution, self-control, and conscientiousness contributed to the risk of being victims of financial fraud. Kadoya et al. (2020a) provided evidence that lack of conscientiousness was an issue contributing to the vulnerability to financial fraud. The Office of Fair Trading (2006) argues that victims of fraud lack cognitive judgment. Holtfreter et al. (2008) argue that lack of self-control contributes to the risk of becoming victims of fraud. Federal Trade Commission (2013) found that risk takers who indulged in risky buying practices were at a higher risk of being victimized. Finally, our results find that respondents who have a feeling of loneliness are more prone to becoming victims of all types of special financial fraud. Feeling lonely is a measure of respondents' sense of isolation, which could occur even if they are living with their family. Lack of emotional and social connectedness leads to loneliness, and thus, lonely people are generally less aware of the possible threat of financial fraud and unable to respond appropriately to fraudsters. Moreover, feelings of loneliness lead to lack of cognitive ability making people psychologically vulnerable. Thus, lonely people are unable to assess financial and commercial transactions, making them susceptible to financial fraud. Findings of the Office of Fair Trading (2006) reveal that socially isolated people are more likely to be victims of fraud, a fact consistent with our argument. Cross (2016) also found that loneliness and isolation among older people make them susceptible to fraud.

### Victims' Profiles at the Aggregate and Specific Levels Using Interaction Variables in the Regression

To gain a deeper insight into the profiles of victims of financial frauds, we used three interaction variables such as gender and marital status, gender and loneliness, and age and loneliness. Table 5 shows the results of the logit regression using interaction variables. The results show that these interaction variables are associated with financial fraud victimization at the aggregate and specific levels. At the aggregate level, the interaction between gender and loneliness has a significantly positive association, while age and loneliness have a significant negative association, indicating that the likelihood of fraud victimization increases when respondents are males and lonely, and younger and lonely, respectively. At the specific level, the interaction between gender and marital status has a significantly negative association, indicating that males are likely to be victims of fictitious billing fraud when they are unmarried. However, the interaction between gender and marital status has a significantly positive association with loan guarantee fraud, indicating that married males are more likely to be victims of loan guarantee fraud. Finally, the interaction between gender and marital status has a significantly positive association, while the interaction between age and loneliness has a significantly negative association with refund fraud, indicating that married males and younger lonely people are more likely to be victims of refund fraud. This result suggests that lonely people, regardless of their age, are likely to be victims of refund fraud.

**Table 5:** Regression results using interaction variables.

**Variable**	**Model 1 (Financial frauds at the aggregate level)**	**Model 2 (Fictitious billing fraud)**	**Model 3 (Loan guarantee fraud)**	**Model 4 (Refund fraud)**
Gender	−0.2609 (−0.75)	0.2618 (0.61)	−1.7061 (−1.99)^**^	−1.9296 (−1.96)^**^
Age	0.0260 (2.85)^***^	0.0147 (1.31)	0.0160 (0.74)	0.0572 (2.30)^**^
Marital status	−0.2851 (−1.50)	0.0044 (0.02)	−0.9651 (−2.40)^**^	−1.0522 (−2.09)^**^
Living with family	0.6820 (4.75)^***^	0.7014 (4.02)^***^	0.7873 (2.23)^**^	0.9534 (2.01)^**^
Education	−0.0032 (−0.14)	−0.0129 (−0.46)	0.0296 (0.56)	−0.0338 (−0.53)
Financial literacy	−0.4650 (−3.45)^***^	−0.4486 (−2.72)^***^	−0.6075 (−1.96)^**^	0.0965 (0.24)
Employment status	−0.0053 (−0.05)	0.0597 (0.44)	0.2487 (0.88)	−0.3300 (−1.04)
Household income	−0.0406 (−1.52)	−0.0425 (−1.25)	−0.0225 (−0.39)	−0.1325 (−1.74)^*^
Household assets	0.0309 (1.58)	0.0070 (0.28)	0.0480 (1.08)	0.0942 (1.80)^*^
Myopic view	−0.0753 (−1.63)	−0.0320 (−0.57)	−0.0951 (−0.89)	−0.1856 (−1.41)
Financial satisfaction	−0.0244 (−0.51)	−0.0986 (−1.66)^*^	0.1637 (1.46)	0.0892 (0.65)
Anxiety	−0.0603 (−1.44)	−0.0429 (−0.82)	−0.1935 (−2.10)^**^	−0.0276 (−0.24)
Careful spending habit	−0.2354 (−5.44)^***^	−0.0921 (−1.66)^*^	−0.5390 (−5.84)^***^	−0.3889 (−3.33)^***^
Trust	−0.0178 (−0.36)	−0.0160 (−0.27)	0.0222 (0.19)	0.1764 (1.26)
Loneliness	0.4162 (3.11)^***^	0.2159 (1.32)	0.3958 (1.27)	0.6599 (1.60)
Gender*married	0.0590 (0.25)	−0.5392 (−1.83)^*^	1.5952 (2.58)^***^	1.7412 (2.39)^**^
Gender*loneliness	0.1555 (1.89)^*^	0.1240 (1.23)	0.1822 (0.94)	0.3045 (1.29)
Age*loneliness	−0.0065 (−2.38)^**^	−0.0030 (−0.87)	−0.0048 (−0.75)	−0.0126 (−1.72)^*^
Cons	−3.1561 (−4.63)^***^	−3.5381 (−4.24)^***^	−3.9571 (−2.52)^**^	−6.2676 (−3.08)^***^
Obs.	11,218	11,189	11,187	11,191
LR Chi^2^	130.04^***^	61.64^***^	90.07^***^	64.64^***^
Pseudo *R*^2^	0.0293	0.0195	0.0795	0.0760
Log likelihood	−2,150.4254	−1,550.2722	−521.5368	−392.6681

*The z values in parentheses*.

***, **, and * represent significance at the 1, 5, and 10% levels, respectively.

## Conclusion

With financial fraud on the rise in Japan, authorities are facing challenges in combating fraudsters because of the innovative swindling methods that they resort to and the changing target groups. Since most people are either unaware of the risks of being swindled or do not report incidents when they have been victims of a scam, identifying people who are at risk and providing protective measures to safeguard them become difficult. Although there have been several studies on profiles of victims of financial fraud at the aggregate level, little or no evidence is found on victim profiles of special financial fraud in Japan, such as fictitious billing fraud, loan guarantee fraud, and refund fraud. This study investigated the demographic, socioeconomic, and psychological backgrounds of victims of special financial fraud in Japan, using a nationwide survey conducted by Hiroshima University, Japan, in 2020. The results of our study revealed that, at the aggregate level, respondents who were male, older, not currently married, living with family, financially illiterate, have a higher balance of financial assets, are concerned about the future, do not have careful buying habits, and are lonely were likely to be victims of special financial fraud. When special financial fraud was disaggregated, the results showed the following: respondents who were male, currently unmarried, living with family, financially illiterate, not satisfied with current financial conditions, do not have careful buying habits, and have a sense of loneliness are generally victims of fictitious billing fraud; respondents who were living with family, financially illiterate, not anxious about life in the old age, do not have careful buying habits, and have a sense of loneliness were likely to be victims of loan guarantee fraud; and respondents who were older, living with family, have a higher balance of financial assets, have low household income, do not have careful buying habits, and have a sense of loneliness are victims of refund fraud. The results of both aggregated and disaggregated financial fraud showed that victims of financial fraud share specific and common characteristics, of which living with family, not having careful buying habits, and loneliness were significant across all types of financial fraud. Results were also indicative of fraudsters' keen ability to identify victims as they targeted specific vulnerable groups for a particular type of fraud.

Our study has important implications for authorities in charge of combating financial fraud. Although creating consumer awareness about financial fraud has been a special agenda for authorities, there is a need to direct the awareness program to vulnerable groups specific to the type of financial fraud. Since lonely people are vulnerable to all kinds of financial fraud, authorities should regularly make them aware of the possible ways in which they could be tricked and teach them to respond appropriately when approached by fraudsters. Additionally, special awareness programs could be directed toward particular groups of people who are likely to be victims of specific financial fraud. For example, older people should be made aware of the possible threat of refund fraud. Our results have important policy implications for the current COVID-19 health pandemic situation as well. With people maintaining social distancing, lonely people, who are generally victims of financial fraud, are at an even higher risk of becoming victims of financial fraud.

Although the study findings suggest directions for authorities to combat financial fraud, it has some limitations as well. First, recognition and reporting bias is likely to exist. Many people who fall victim to financial fraud either do not recognize the issue as fraud or do not report the fraud, to avoid social distress and embarrassment. Thus, special financial fraud discussed in this study could be underreported. Nevertheless, this underreporting does not materially affect the results of because of the large sample size and the assurance of anonymity given to respondents. However, this issue should be considered while interpreting the results. Second, the potential causal relationship between loneliness and financial fraud victimization could influence our results. Although we are aware of the issue, we cannot control it due to limitation of data. Future studies should take further measures for respondents to freely disclose incidents of financial fraud. We suggest focusing on older and lonely people who have been facing increasing challenges during this health pandemic. Additionally, along with socioeconomic and psychological backgrounds, future research should focus on the circumstances through which fraudsters trap victims.

## Data Availability Statement

The raw data supporting the conclusions of this article will be made available by the authors, without undue reservation.

## Ethics Statement

The ethical review and approval were not necessary according to institutional requirements (the ethics committee of Hiroshima University). The patients/participants provided their written informed consent to participate in this study.

## Author Contributions

YK and MK: study design. YK, MK, JN, and SW: analysis and interpretation of data and writing of the report. All authors contributed to the article and approved the submitted version.

## Conflict of Interest

The authors declare that the research was conducted in the absence of any commercial or financial relationships that could be construed as a potential conflict of interest.
