# Supplementary material for: Who Is Next? A Study on Victims of Financial Fraud in Japan
Source: Front Psychol. 2021 Jul 2;12:649565. doi: 10.3389/fpsyg.2021.649565 (PMC8283193; doi:10.3389/fpsyg.2021.649565)
Supplement: Supplementary file 1 [file Data_Sheet_1.PDF]

Table Test of multicollinearity (Variance Inflation Factor)

Variable	VIF	1/VIF
Assets	1.710	0.586
Income	1.610	0.621
Age	1.600	0.626
Employment status	1.480	0.677
Gender	1.470	0.678
Financial satisfaction	1.390	0.718
Living with family	1.350	0.738
Married	1.310	0.763
Anxiety	1.300	0.768
Financial literacy	1.270	0.788
Education	1.190	0.838
Loneliness	1.130	0.887
Buying behavior	1.120	0.895
Trust	1.100	0.911
Future	1.090	0.921
Mean VIF	1.340	

Table Correlation matrix

	gender	age	married	livefamily	educ	finliteracy	emplstatus	income	assets	future	finsatis	anxiety	buybehav	trust	lonely
gender	1.00														
age	0.34*	1.00													
married	0.03*	0.05*	1.00												
livefamily	0.00	0.13*	0.40*	1.00											
educ	0.18*	-0.02*	0.07*	-0.04*	1.00										
finliteracy	0.29*	0.23*	0.04*	0.01	0.26*	1.00									
emplstatus	0.31*	-0.22*	0.15*	-0.11*	0.19*	0.10*	1.00								
income	0.10*	0.02*	0.32*	0.30*	0.24*	0.17*	0.26*	1.00							
assets	0.14*	0.37*	0.09*	0.13*	0.22*	0.29*	-0.04*	0.42*	1.00						
future	-0.05*	-0.07*	-0.05*	-0.06*	-0.10*	-0.17*	-0.01	-0.09*	-0.15*	1.00					
finsatis	0.04*	0.14*	0.13*	0.11*	0.12*	0.10*	-0.01	0.27*	0.38*	0.01	1.00				
anxiety	-0.14*	-0.15*	-0.04*	-0.03*	-0.09*	-0.07*	-0.01	-0.16*	-0.27*	-0.01	-0.37*	1.00			
buybehav	-0.03*	0.02*	-0.02*	0.03*	0.02*	0.13*	-0.05*	-0.02	0.04*	-0.16*	-0.01	0.22*	1.00		
trust	0.04*	0.18*	0.07*	0.06*	0.04*	0.04*	-0.04*	0.06*	0.10*	0.06*	0.23*	-0.09*	-0.02*	1.00	
lonely	-0.05*	-0.16*	-0.13*	-0.15*	-0.03*	-0.04*	0.03*	-0.13*	-0.11*	0.06*	-0.23*	0.21*	-0.03*	-0.15*	1.00

* Represents significance at least at the 5% level.
